# COVID-19-Induced Rheumatoid Arthritis: Case Series and Systematic Review of the Literature

**DOI:** 10.31138/mjr.171223.cir

**Published:** 2024-05-21

**Authors:** Selma Bouden, Hiba Ben Ayed, Leila Rouached, Aicha Ben Tekaya, Ines Mahmoud, Raoudha Tekaya, Olfa Saidane, Leila Abdelmoula

**Affiliations:** Rheumatology Department, Charles Nicolle Hospital, Tunis, Tunisia

**Keywords:** COVID-19, rheumatoid arthritis, antibodies, case report

## Abstract

COVID-19, caused by a severe acute respiratory syndrome coronavirus 2 (SARS-CoV-2), can lead to severe infection and has been suggested to induce autoimmune phenomena. We report three cases of rheumatoid arthritis (RA) occurring after COVID-19 infection and we present a systematic review of the literature of cases of RA post COVID-19. Our findings suggest that RA may be trigged by COVID-19 infection in genetically predisposed individuals.

## INTRODUCTION

The Coronavirus disease 2019 (COVID-19) has since spread rapidly across the world. Since being first discovered in China, multiple waves of outbreaks have been reported in many countries. Some studies suggest that the virus can cause uncontrolled immune activation and cytokine responses, leading to the manifestations of a variety of diseases, including autoimmune rheumatic manifestations.^1,2^ Although most musculoskeletal symptoms caused by the SARS-CoV-2 infection are usually acute and self-limiting, chronic arthritis is rarely observed.^2,3^ Recent research suggests there may be a link between COVID-19 and the development of rheumatoid arthritis (RA).^1,4^ Indeed, the pattern of proinflammatory cytokines induced in COVID-19 appears to be similar to that in RA, suggesting similar disease mechanisms.^1^ To the best of our knowledge, only 11 cases of RA-compatible polyarthritis following COVID-19 infection have been reported.^5–12^ Herein, we report three patients with chronic inflammatory arthritis consistent with post COVID-19 RA and we realise a systematic review of literature of cases of RA post COVID-19.

## SEARCH STRATEGY

The systematic review was performed based on the Preferred Reporting Item for Systematic Review and Meta-analysis (PRISMA) guidelines. We searched the following online databases (PubMed, Embase, Scopus, and Web of Science) for studies up to March 2023 using the following keywords (“COVID-19”[Mesh] OR “SARSCoV-2”[Mesh]) AND (“Rheumatoid Arthritis, Systemic Juvenile” [Supplementary Concept] OR “Arthritis, Rheumatoid” [Mesh]). No restrictions were imposed. We also did manual research on reference lists of retrieved relevant articles.

## STUDY SELECTION

In this review, we included prospective or retrospective descriptive case reports and case series conducted on post-COVID 19 RA. We excluded papers investigating other post-COVID chronic inflammatory rheumatic diseases and papers not published in English or French.

## DATA EXTRACTION

The following data were collected from each included article: age of cases, gender, date of COVID-19 infection, COVID-19 infection severity (based on the United States Centres for Disease Control and Prevention definition of severe outcomes of COVID-19 as hospitalisation, admission to the intensive care unit (ICU), intubation or mechanical ventilation, or death,^13^), date of the joint symptoms onset, C-reactive protein (CRP), rheumatoid factor (RF) and Anti-citrullinated protein antibodies (ACPA) positivity, treatment, and treatment outcomes.

## CASE PR ESENTATION

### Patients’ information

This is a report of the case of three women who were diagnosed with RA according to the 2010 American Congress of Rheumatology/European League Against Rheumatism (ACR/EULAR) criteria after recovery from the COVID-19 infection. The first patient was 27 years old, the second patient was 55, and the third patient was 62. A family history of inflammatory arthritis was found in one patient. All patients were non-smokers and none of them had a prior autoimmune disease. COVID-19 infection was non-severe in two patients. The three patients have not received the COVID-19 vaccination and all of them presented to our department with inflammatory bilateral arthralgia of large and small joints after recovery from the COVID-19 infection.

The time between COVID-19 infection and symptoms onset was of 16 weeks in patient 1 and of 12 weeks in patients 2 and 3. The time between joint symptoms onset and RA diagnosis was of eight weeks in patients 1 and 3 and of two weeks in patient 2.

### Clinical Findings

Physical examination revealed bilateral symmetric arthritis. Other rheumatologic manifestations (except for arthritis) were found in only one patient who also complained of chronic myalgia after COVID-19 infection and in whom muscle enzyme levels were normal. All patients denied a history of chronic low-back pain, Raynaud’s phenomenon, rash, dry eyes and mouth, and diarrhoea. The lower lumbosacral spine and sacroiliac region examinations were without pathological findings. Analysis of the synovial fluid aspirate of the knees was performed in one patient and was consistent with inflammatory arthritis with yellow fluid, a total nucleated cell count (TNCC) of 3000 cells/mm3 (69% neutrophils), no red blood cells (RBCs) or crystals, and negative growth on bacterial culture.

Clinical details of patients and arthritis characteristics are summarised in **Table 1**.

**Table 1.** Demographic, clinical, and biological characteristics of the patients with new-onset rheumatoid arthritis after COVID-19 infection

**Patient characteristics**		**Family history of inflammatory arthritis/autoimmune diseases**	**Patient’s medical history**	**Smoking**	**Date of COVID-19 infection**	**COVID-19 infection severity**	**Other rheumatologic manifestations**	**Date of the joint symptoms onset**	**Time between COVID-19 infection and symptoms onset**	**Time between joint symptoms onset and RA diagnosis**	**Number of tender joints at the time of RA diagnosis**	**Number of swollen joints at the time of RA diagnosis**	**CRP (mg/L)**	**RF (U/mL)**	**ACPA (U/mL)**
**Age**	**Sex**														
27 years	Female	No	No	No	July 2022	Paucisymptomatic	Myalgia	October 2022	16 weeks	8 weeks	15	13	53	62 (N <12)	192 (N<5)
55 years	Female	Yes (RA history in two sisters)	Hypertension Hypercholesterolemia Ischemic heart disease Cerebrovascular Accident	No	November 2022	Moderate (had a seven-day hospital admission without need for intensive care)	No	January 2023	12 weeks	2 weeks	10	9	54.3	45.9 (N<12)	Not tested
62 years	Female	No	Hypertension	No	December 2021	Paucisymptomatic	No	February 2022	12 weeks	8 weeks	8	6	23	142 (N <12)	104 (N<5)

*RA= rheumatoid arthritis; CRP=C-reactive protein ; RF=rheumatoid factor ; ACPA=Anti-citrullinated protein antibodies*

### Diagnostic Assessment

CRP was high in all patients with a mean of 40.3 mg/l.

Complete blood count and creatinine were normal. RF was strongly positive (>3-fold higher than the upper normal range) in all cases. ACPA were tested in two patients and were also strongly positive (192 U/mL and 105 U/mL, normal < 5 U/mL). Antinuclear antibodies (ANA) were positive and speckled in all patients, ranging from 1/100 to 1/400.

**Figure 1.**
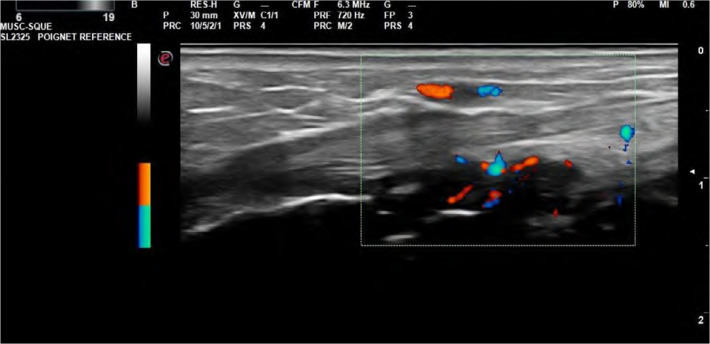
Ultrasonography of the hands showing positive Doppler synovitis (grade 3) of the right wrist.

**Figure 2.**
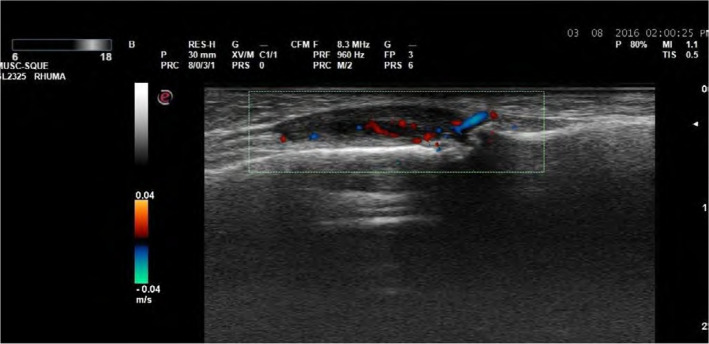
Ultrasonography of the second left intercarpal joint showing positive Doppler synovitis (grade 3).

In all patients, X-rays of the wrists and hands showed periarticular osteopenia. Joint ultrasound examination detected ankle synovitis and tenosynovitis in patient 1, wrist synovitis with a Power Doppler signal in patient 2, and bilateral synovitis in the radio-ulnar joint, radio-carpal joint, second and third left intercarpal joints, and radio-humeral joints with an active Doppler signal in patient 3 (**Figures 1** and **2**).

### Therapeutic Intervention

Upon RA diagnosis, all the patients were started on corticosteroids therapy and 15 mg/week of methotrexate (MTX). Patients 2 and 3 experienced significant improvement shortly after the initiation of the treatment so that prednisone dose was tapered to a maintenance dose of 5 mg/day for three months. Patient 2 achieved low disease activity after six months of MTX initiation, as assessed by a 28-disease-activity-score (DAS28) of 3.1. However, an increase in the MTX dose to 20 mg/week was needed in patient 3 as the DAS28 at the 3-month follow-up visit was of 4.82.

### Follow-up and Outcomes

For Patient 1, we are currently at two-month decline in treatment, only complains of minimal discomfort on his wrists and hands with no relapse of the swelling.

### Discussion and Review of the Literature

In the present study, we report the clinical, laboratory and imaging findings of three patients with chronic inflammatory arthritis consistent with post COVID-19 RA and we realise a systematic review of the literature of cases of RA post COVID-19. To the best of our knowledge, only 11 new cases of RA have been reported following the new pandemic COVID-19.^5–12^ (**Table 2**). We reviewed the literature in order to identify cases of post-COVID19 RA. Seven articles were eligible. Eleven cases were reported in the literature.^5–12^ (**Table 2**).

**Table 2:** Cases of post-COVID 19 rheumatoid arthritis reported in the literature.

**Authors year**	**Number of cases**	**Age / gender**	**COVID-19 infection severity**	**Time from infection to joint symptoms onset**	**CRP (mg/L)/ESR (mm/h)**	**RF (U/mL)/ACPA (U/mL)**
Derksen et al.^5^ 2021	Four	NM	Moderate-to-severe	6.6 weeks	NM	RF: NM ACPA positive in 2 patients
Tamborrini et al.^8^ 2021	One	36 M	Moderate	6 weeks	Normal/Normal	RF: positive (299) ACPA: positive (146)
Perrot et al.^9^ 2021	One	60 F	Moderate	25 days	18.5 NM	RF : Negative ACPA :Positive
Baimukhamedov et al.^10^ 2021	One	67 M	Severe	37 days	55 59	RF: positive (411) ACPA: positive (104)
Roongta et al.^11^ 2021	One	56 M	Severe	14 days	Elevated CRP and ESR	RF: positive (131) ACPA: (35)
Deeb et al.^6^ 2022	One	63 M	Severe	2 months	113	RF: positive (133) ACPA: positive (64)
Slouma et al.^7^ 2022	One	72 F	Asymptomatic	2 weeks	108 95	RF: positive (128) ACPA: positive (200)
Bouzid et al.^12^ 2022	One	38 F	Moderate	1 month	4.3/13	RF : positive (62.29) ACPA: positive (237.39)
Our cases	Three	3 F, mean age 48 years	Pauci-symptomatic to moderate	3.3 months [3–4 months]	Mean CRP: 43.4 NM	RF: positive in all patients ACPA: tested in 2 patients/positive

NM: not mentioned; CRP: C-reactive protein; RF: rheumatoid factor; ACPA: Anti-citrullinated protein antibodies.

Previous publications have highlighted the important role of viral infections in the development of autoimmune diseases.^2^ Examples include type 1 diabetes, post-influenza IgA vasculitis, and antiphospholipid antibody syndrome,^14^ as well as Zika virus,^15^ chikungunya,^15^ hepatitis C, parvovirus B19, rubella, and hepatitis Transverse myelitis, arthralgia, myalgia, and arthritis following B virus.^16^ COVID-19 does not typically cause clinical arthritis but rather myalgia and arthralgia,^2,4^ which occur in 10% to 15% of patients with COVID-19 infection.^3,4,17^ Reactive arthritis appears to be the most common form of chronic arthritis in post-COVID patients. In fact, in a recent Egyptian study involving 100 COVID-19 patients who recovered 6 months ago, the prevalence of reactive arthritis was 37%, with a significant association with old age (p=0.01), smoking (p=0.001) and arthralgia (p=0.049).^18^ The duration between infection and arthritis onset was not reported in this study, but in a retrospective study in India of 23 patients who developed reactive arthritis after infection with COVID-19, the duration was 25.9 days (range 5 to 52 days).^19^ A case of COVID-19-induced psoriatic arthritis was also recently reported in a 51-year-old man who developed joint symptoms 10 days after recovery and showed clinical improvement after treatment with a TNF-α inhibitor.^20^

The link between human coronaviruses and the risk of RA is not new and goes back to before the pandemic. This was well described in a large Korean study.^21^ In this study, weekly RA incident data (2012–2013) were obtained from the National Health Insurance Claims Database, and weekly coronavirus infection observation data were obtained from the Korean Centres for Disease Control and Prevention database.^21^ The percentage change in incident RA associated with ambient mean respiratory viral infections was assessed using a generalised linear model after adjusting for time trends, air pollution, and meteorological data. A total of 24,117 cases of incident RA were analysed. Ambient coronavirus infection in the population was associated with a higher number of incident RA over time, and its effect peaked 6 or 7 weeks after exposure.21 In fact, coronavirus was associated with an increase in the number of incident RA of 9.2% (from 3.9 to 14.8, p<0.001) with an incremental increase of 1% in virus detection rate. (p<0.001).^21^

Derksen et al. aimed to investigate the association between preceding COVID-19 infection and new-onset RA among 61 post-COVID patients.^5^ The mean age of patients was 63.6 years. Five patients were hospitalised due to severe COVID-19 and joint complaints started on average 6.6 weeks after infection.^5^ Four out of 5 patients fulfilled the ACR 2010 criteria for RA. Three patients were phenotypically very similar to regular new-onset RA patients, and one patient had a history of seronegative RA and had been in DMARD-free remission for 5 years.^5^ In Slouma et al.^7^ study, which also reported a case of RA post COVID19 infection, the human leukocyte antigen (HLA) class II genotyping was performed and showed the presence of the alleles: HLA-DRB1*04:11, HLADQB1*03:01, and HLA-DQB1* 03:02.

Several theories have been proposed about how people with a genetic predisposition might develop RA after contracting COVID-19. One proposed mechanism involves molecular mimicry.^22^ The coronavirus epitope (spike glycoprotein S) is similar to human epitopes and plays a key role in host cell invasion and evasion of immune response attack. These epitopes are present in multiple tissues of the body, including neural tissue and synovium, and may be responsible for a variety of organ-specific diseases.^18^

This may also lead to subsequent proliferation of autoantibody-producing B cells, including those that produce rheumatoid factor and cytokines, as seen in Epstein-Barr virus-induced RA.^23^

In addition to molecular mimicry, (hyper)inflammation appears to be a viable mechanism by which COVID-19 induces autoimmunity. In fact, the pattern of pro-inflammatory cytokines induced in COVID-19 is similar to target cytokines for RA treatment; Several studies have showed that primary epithelial cells are infected with COVID-19, serve as a source of cytokines, and secrete TNF-α., IL-6, IL-1 and IL-8. 24 In this sense, Taha et al.^18^ showed that pre-treatment IL6 level was a significant predictor of post-COVID 19 arthritis with a difference of 3.988 (p = 0.007). However, no significant relationship was observed with serological markers of autoimmunity (ANA RF and ACPA).^18^ Additionally, in the study of Derksen et al.,^5^ the authors evaluated the prevalence of ACPA after COVID-19 by measuring ACPA IgG in 61 patients and concluded that the seroprevalence of this antibody did not increase after COVID. All these findings support the fact that the underlying mechanism of post-COVID-19 arthritis is most likely due to the hyperinflammatory process associated with COVID-19 infection rather than an autoimmune response. However, a parainfectious unmasking of an underlying dormant RA in a genetically predisposed individual is defendable. Indeed, a genome-wide association study in COVID-19 patients found that locus 3p21.^31^ was associated with disease severity.^25^ Interestingly, this region contains the lymphokine-1 and -2 receptor XCR1, which is involved in antigen cross-presentation and has been reported in the RA synovium.^26^

We admit some limits of our study, mainly the retrospective method design and the small number of cases which makes them difficult to analyse and, therefore, draw more precise conclusions.

To conclude, we described three cases of RA new-onset occurring 12 to 16 weeks after recovery from COVID-19 infection. The link between the virus and RA is difficult to establish. However, it seems that RA may be trigged by an inflammatory activation of the immune system in genetically predisposed individuals.

## Data Availability

The authors declare that the data supporting the findings of this study are available within the article and its supplementary material. Raw data that support the findings of this study are available from the corresponding author, upon reasonable request.
